# Antibody-independent functions of B cells during viral infections

**DOI:** 10.1371/journal.ppat.1009708

**Published:** 2021-07-22

**Authors:** Vinit Upasani, Izabela Rodenhuis-Zybert, Tineke Cantaert

**Affiliations:** 1 Immunology Unit, Institut Pasteur du Cambodge, Institut Pasteur International Network, Phnom Penh, Cambodia; 2 Department of Medical Microbiology and Infection Prevention, University of Groningen and University Medical Center Groningen, Groningen, the Netherlands; Yale University School of Medicine, UNITED STATES

## Abstract

The humoral immune response and antibody-mediated functions of B cells during viral infections are well described. However, we have limited understanding of antibody-independent B cell functions, such as cytokine production and antigen presentation, in acute and chronic viral infections and their role in protection and/or immunopathogenesis. Here, we summarize the current literature on these antibody-independent B cell functions and identify remaining knowledge gaps. B cell subsets produce anti- and pro-inflammatory cytokines, which can have both beneficial and detrimental effects during viral clearance. As professional antigen presenting cells, B cells also play an important role in immune regulation/shaping of the developing adaptive immune responses. Since B cells primarily express TLR7 and TLR9, we specifically discuss the role of Toll-like receptor (TLR)-mediated B cell responses to viral infections and their role in augmenting adaptive immunity through enhanced cytokine production and antigen presentation. However, viruses have evolved strategies to subvert TLR signaling and additional stimulation via B cell receptor (BCR) may be required to overcome the defective TLR response in B cells. To conclude, antibody-independent B cell functions seem to have an important role in regulating both acute and chronic viral infections and may form the basis for novel therapeutic approaches in treatment of viral infections in the future.

## Introduction

**Fig 1:**
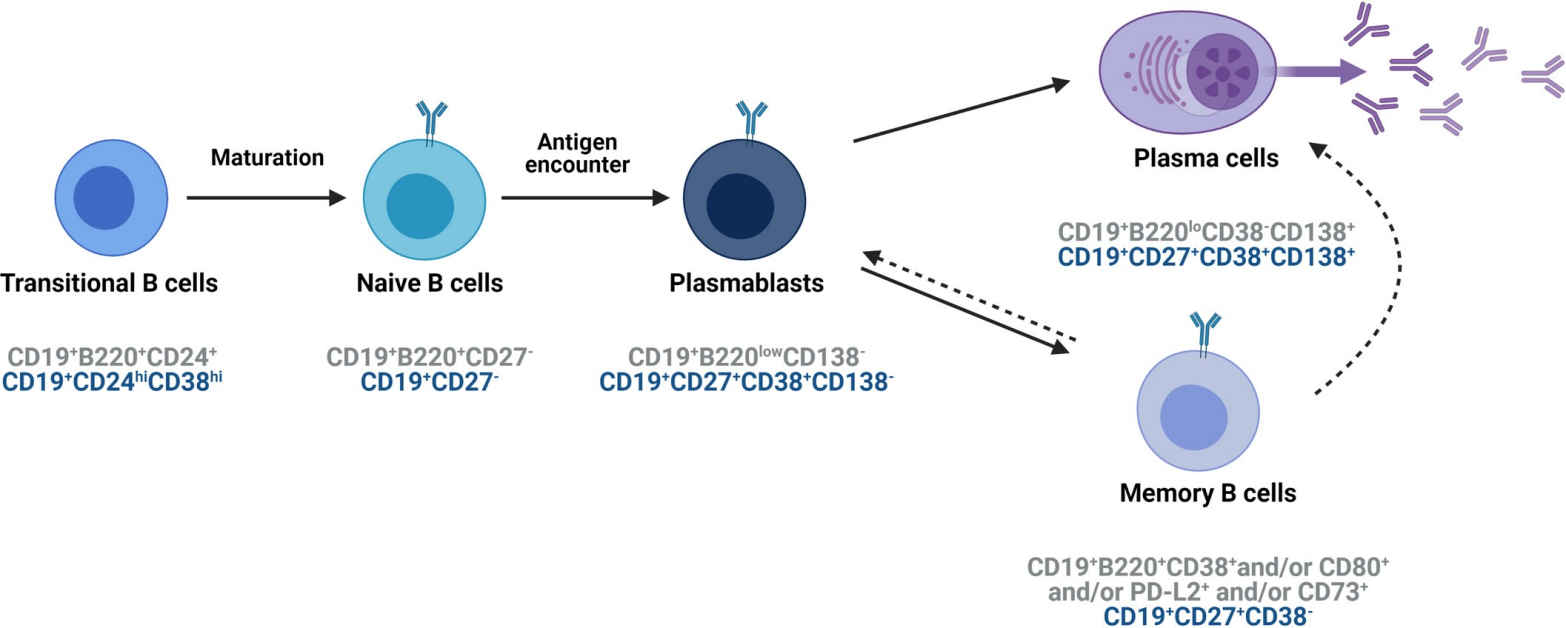
Summary of B cell subsets in mice and humans. Stages of B cell maturation and the expression of cell-specific markers for these B cell subsets in mice (grey) and humans (blue). Created with BioRender.com.

B cells are derived from hematopoietic stem cells in the bone marrow. After exiting the bone marrow, immature new emigrant B cells (murine: CD19^+^B220^+^CD24^+^; human: CD19^+^CD24^hi^CD38^hi^) circulate in the peripheral compartment and mature further into naive B cells (murine: CD19^+^B220^+^CD27^−^; human: CD19^+^CD27^−^). Via their B cell receptor (BCR), B cells recognize native, unprocessed antigens present on the surface of pathogens, in soluble form or captured by follicular dendritic cells (DCs). Upon recognition of the antigen, B cells are activated and differentiate into memory cells (murine: CD19^+^B220^+^CD38^+^ and/or CD80^+^ and/or PD-L2^+^ and/or CD73^+^ and human: CD19^+^CD27^+^CD38^−^, CD19^+^CD27^−^IgG^+^), short-lived antibody-secreting plasmablasts (murine: CD19^+^B220^low^CD138^+^; human: CD19^+^CD27^+^CD38^+^CD138^−^), and long-lived plasma cells (murine: CD19^+^B220^low^CD27^+^CD38^−^CD138^+^; human: CD19^+^CD27^+^CD38^+^CD138^+^), all of which are recognized as the conventional effector cells involved in the humoral immune response (Fig 1). Besides their important role in mediating humoral immunity, B cells have been shown to exert antibody-independent functions. Upon stimulation via BCR, Toll-like receptor (TLR), and/or CD40L, human and mouse B cell subsets become activated and can secrete both anti-inflammatory cytokines such as interleukin (IL)-10, IL-35, and TGF-β and pro-inflammatory cytokines such as IL-6 and tumor necrosis factor alpha (TNF-α) [1–7]. These cytokines, in turn, influence effector T cell responses [1–7] and contribute to the development and suppression of autoimmunity in both humans and murine models [8–10]. Furthermore, B cells upon BCR ligation and IL-21 stimulation secrete the cytotoxic molecule granzyme B (GrB), which can play a significant role in early antiviral immune responses and in the regulation of autoimmune responses [11]. B cells also have a direct impact on T cell functionality as they can serve as antigen-presenting cells (APCs), thereby enhancing T cell–mediated responses [12,13] and can provide costimulatory signals to T cells [14,15]. In addition, human B cells express TLRs such as TLR7 and TLR9, which are known to recognize viral ligands. In B cells, sensing of ligands by TLRs promotes B cell activation, antigen presentation, proliferation, class switch recombination, and antibody secretion [16,17].

The function of B cells as precursors of antibody-secreting cells and of antibodies in the immune response to viral infection is extensively studied, but little is known about antibody-independent B cell responses. We have limited understanding of the roles of B cells during the acute and chronic phase of infection and their contribution to protection of infection or immunopathogenesis. Hence, the current review aims to summarize the known antibody-independent functions of B cells during viral infections and identifies gaps in our knowledge on B cell function during both acute and chronic viral infections.

### IL-10–producing regulatory B cells during viral infection

**Fig 2:**
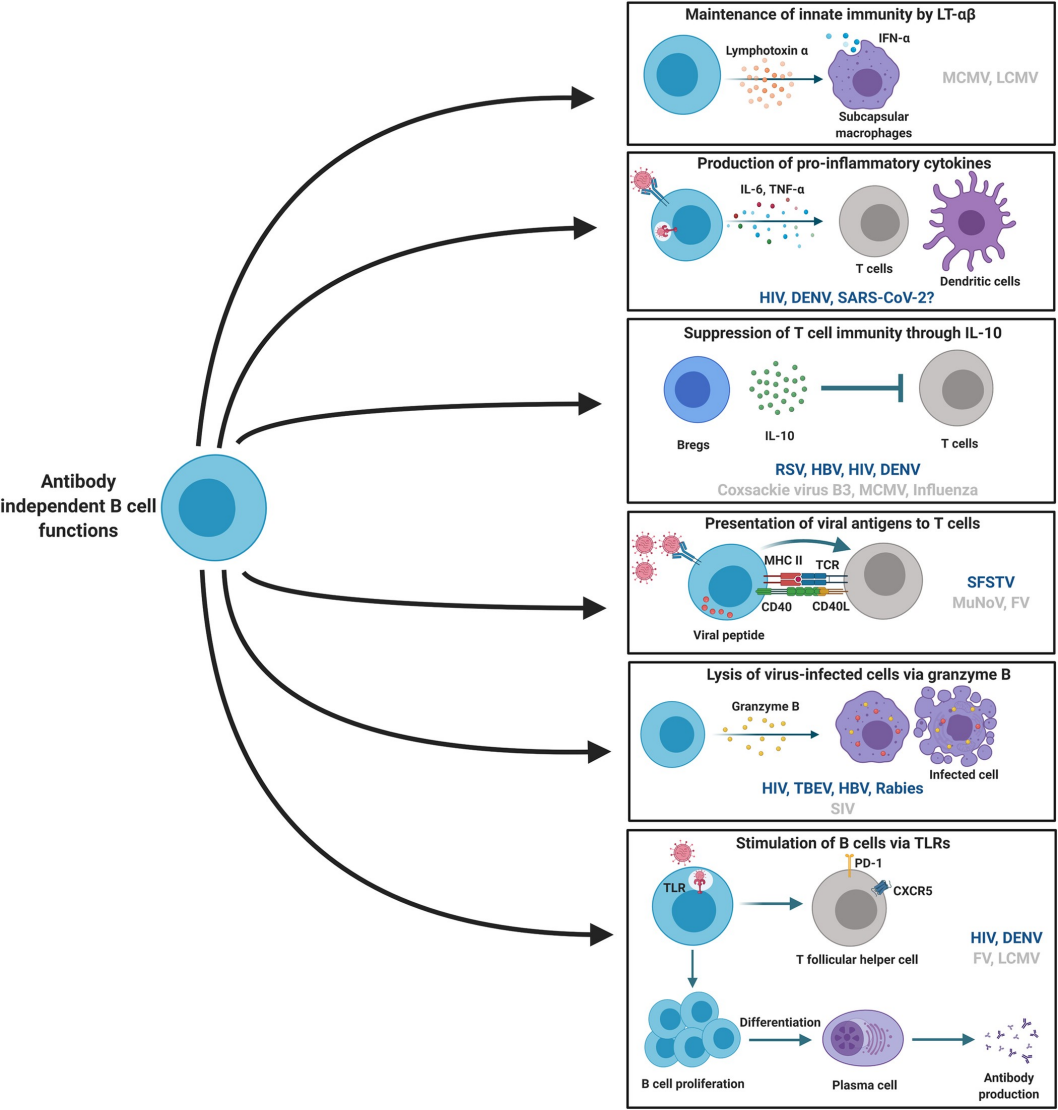
Summary of antibody-independent B cell functions in viral infections. B cells influence immune response to viral infections through production of pro-inflammatory and regulatory cytokines. B cells produce other effector molecules such as cytotoxic GrB and LT-α, which mediate various immune functions. Granzyme B (GrB^+^) B cells may contribute to apoptosis of virus-infected cells. B cells produce LT-α, which enhances type I interferon production in myeloid cells. B cells act as professional APCs by presenting viral peptides via MHC II to CD4^+^ T cells. Stimulation of B cells via TLRs induces downstream signaling resulting in production of antibodies. Created with BioRender.com. APC, antigen-presenting cell; GrB, granzyme B; DENV, dengue virus; FV, Friend virus; HBV, hepatitis B virus; LCMV, lymphocytic choriomeningitis virus; LT-α, lymphotoxin-α; MCMV, murine cytomegalovirus; MHC II, major histocompatibility complex class II; MuNoV, murine norovirus; RSV, respiratory syncytial virus; SARS-CoV-2, severe acute respiratory syndrome coronavirus 2; SFSTV, severe fever with thrombocytopenia syndrome virus; SIV, simian immunodeficiency virus; TBEV, tick-borne encephalitis virus; TCR, T cell receptorTLR, Toll-like receptor.

The immune system has evolved multiple feedback mechanisms for regulating the initiation, activation, and contraction phase of the immune response to foreign antigens, thereby providing a well-balanced protective immune response. One of these mechanisms relies on IL-10–mediated pathways. IL-10 regulates and suppresses the expression of pro-inflammatory cytokines during the recovery phases of infections and is considered a master negative regulator of inflammation [18,19]. Blockade in the IL-10 pathway typically results in prolonged and exaggerated immune responses to antigens that can lead to immunopathology [19]. IL-10 is produced by a variety of innate and adaptive immune cells, including macrophages, DCs, natural killer (NK) cells, CD4, CD8, γδ T cells, and various B cell subsets. IL-10–producing regulatory B cells (Bregs) display immunoregulatory functions in both humans and mice. These cells have been the subject of an increasing body of work in recent years. Bregs encompass a variety of B cell subsets, all of which are defined by their ability to produce IL-10 and/or other immunoregulatory cytokines [20]. The B cell subsets that produce IL-10 in vivo have long been debated, and in vitro studies have yielded conflicting results. In mice, transitional 2 marginal-zone precursor (T2-MZP) cells, CD5^+^CD1d^hi^ B (B10) cells, marginal-zone (MZ) B cells, Tim-1^+^ B cells, CD138^+^ plasma cells, and plasmablasts have been suggested to contain IL-10–producing Bregs [20]. Using an IL-10 reporter mouse system, Matsumoto and colleagues have shown that mainly plasmablasts expressed IL-10 in a model of experimental autoimmune encephalomyelitis (EAE) and that removal of these cells exacerbated the disease [21]. In humans, IL-10–producing Bregs have been identified as CD19^+^CD24^hi^CD38^hi^CD1d^hi^ and CD19^+^CD24^hi^CD27^+^ [11]. However, there is no specific lineage marker or transcription factor identified in human or mouse that discretely identifies Bregs. Whether the phenotypic differences observed are due to the existence of distinct Breg lineages or to changes dependent upon the immunological environment has yet to be elucidated. One hypothesis is that the inflammatory milieu could stimulate the development of Bregs [21]. Involvement of IL-10–producing Bregs is largely beneficial in protection from excessive immune activation during autoimmune diseases, tumor development, and transplantation; however, they may also contribute to the aggravation of disease by suppressing beneficial T cell–mediated responses [22,23]. It has been demonstrated that B cell–derived IL-10 can modulate the inflammatory responses during viral infections [Table 1]. IL-10 can potentiate both the detrimental and beneficial effects of inflammation and can even contribute to pathogenesis of both acute and chronic viral infections [Fig 2].

**Table 1:** This table shows currently described subsets of Bregs and IL-10–producing B cells during viral infections in humans and mice.

**Human B cells defined as Bregs and/or B cells producing IL-10**			
**Identified Phenotype**	**Virus**	**Key Features**	**Reference**
Immature/transitional cells (Bregs) (CD19^+^CD24^hi^CD38^hi^)	HBV	Increased frequencies correlated temporally with hepatic flares, both after stimulation and directly ex vivo. Involved in regulation of antigen-specific CD8+ T cells in HBV infection	[23]
CD19^+^IL-10^+^			[24]
Immature/transitional cells (Bregs)(CD19^+^CD24^hi^CD38^hi^)	HIV	Reduced frequencies found in HIV-infected individuals, suppress CTL functions by activated Bregs, which causes viral persistence	[25, 26]
Immature/transitional cells (Bregs)(CD19^+^CD24^hi^CD38^hi^)	Dengue	Reduced frequencies correlated with development of severe dengue. When isolated from dengue patients produced negligible quantities of IL-10 upon stimulation in vitro	[35]
Neonatal Bregs (CD5^hi^CD10^−^CD1c^lo^CD21^int^CD45RA^int^CD23^hi^CD24^lo^CD38^lo^IgD^lo^IgM^lo^)	RSV	Neonatal Bregs produce IL-10 that dampens beneficial cytokine production by Th1 cells and contributes to severe disease.	[22]
**Murine B cells defined as Bregs and/or B cells producing IL-10**			
**Phenotype**	**Virus**	**Key Features**	**Reference**
B10 cells (CD19^+^CD1d^hi^CD5^+^)	MCMV	Bregs via IL-10 modulate T lymphocyte as well as microglial cell responses within the infected mice brain and promote CD4^+^Foxp3^+^ T-cell proliferation in vitro.	[30]
CD220^lo^CD1a^+^ and CD220^hi^CD1d^+^	Influenza	Toso-specific antibodies selectively induced IL-10–competent B cells at the site of inflammation; decreased pro-inflammatory cytokine production by lung T cells in influenza A–infected mice	[34]

Bregs, regulatory B cells; CTL, cytotoxic T lymphocyte; HBV, hepatitis B virus; IL-10, interleukin 10; MCMV, murine cytomegalovirus; RSV, respiratory syncytial virus.

#### Potential detrimental effects of IL-10–producing Bregs on antiviral T cell responses

Bregs producing IL-10 has been shown to exert pleiotropic unfavorable effects in the course of virus infections and some of them have been linked to severe disease pathogenesis. In the course of acute respiratory syncytial virus (RSV) infection in infants, infected neonatal Bregs produce IL-10 that dampens beneficial cytokine production by Th1 cells and contributes to severe disease [24]. In patients with chronic hepatitis B infection, increased frequencies of immature B cells (CD19^+^CD24^hi^CD38^hi^) were shown to suppress beneficial antiviral hepatitis B virus (HBV)-specific CD8^+^ T cell responses in vitro in an IL-10–dependent manner and were thus implicated in the pathogenesis of disease, including the development of hepatic flares [25,26]. In addition, CD19^+^CD24^hi^CD38^hi^ B cells from HBV patients were able to inhibit IFN-γ production by CD4^+^ T cells and induce the formation of IL-10–producing Tregs [26]. In another cohort, the frequencies of IL-10–producing B cells and serum IL-10 concentrations correlated positively with flare-ups of liver disease in patients with chronic hepatitis B infection [27]. Further studies using in vivo models are necessary to elucidate a causal relationship between frequencies of Bregs and HBV disease status. Increased frequencies of IL-10–producing Bregs and B cell–derived IL-10 have been reported in HIV-infected individuals compared to HIV–negative individuals [28–30]. Upon stimulation in vitro with TLR ligands and CD40L, these Bregs suppressed protective CD8^+^ T cell responses and may have inadvertently contributed to the persistence of virus in these patients [28]. Moreover, blockade of the IL-10 pathway augmented in vitro proliferation of HIV-specific CD4^+^ and CD8^+^ T cells and increased cytokine secretion by HIV-specific CD4^+^ T cells [29]. Overall, these studies highlight the capacity of IL-10–producing B cells to regulate antigen-specific CD8^+^ T cells in humans and implicate these cells in chronic HBV and HIV pathogenesis.

The role of IL-10–producing Breg cells has been further elucidated in mice models of acute and chronic viral infection. In mice infected with murine cytomegalovirus (MCMV), which causes chronic infection, B cells in lymphoid tissues up-regulate the expression of IL-10, which was shown to decrease virus-specific CD8^+^ T cell responses and expansion of plasma cells [31]. Indeed, knockout of IL-10 in B cells resulted in increased frequencies of cytotoxic CD8^+^ T cells when compared to wild-type mice and resulted in better virus clearance [31]. IL-10–producing Breg cells were shown to infiltrate the brains of mice chronically infected with MCMV [32]. In fact, herpesviruses have evolved multiple strategies to promote the production of IL-10, which, by dampening beneficial to the host T cell–mediated immunity, facilitates the establishment of persistent virus infections [33,34]. For example, the M2 protein of murine herpesvirus drives the proliferation and survival of IL-10–expressing B cells, which are capable of suppressing antiviral T cell immunity [35].

#### Potential beneficial responses of Bregs during viral infection

Notably, IL-10 produced by B cells can also have beneficial effects during infections by suppressing excessive T cell activation and dampening inflammation. These effects are mainly apparent during acute viral infection. For instance, in influenza virus infection models, treatment with antibodies binding to Toso (FcμR or FcR for IgM) on B cells induced the differentiation of Toso-deficient B cells into IL-10–competent B cells. These cells migrated to the lungs at the site of influenza-induced inflammation and secreted IL-10, thereby decreasing pro-inflammatory cytokine production by lung T cells [36]. However, the role of Bregs in response to MCMV, which can cause chronic, persistent infections in mice, is not clear. In the absence of IL-10–producing B cells during MCMV infection, one study has shown an increase of virus-specific CD8^+^ T cells and increased viral clearance [31]. However, another study has shown that the absence of IL-10–producing Bregs contributed to exacerbated neuroimmune responses, suggesting a protective role by these cells [32]. Furthermore, in our study on B cell responses in dengue patients, we observed higher frequencies of CD19^+^CD24^hi^CD38^hi^ B cells, which are known to produce IL-10, in patients with mild dengue compared to those with severe dengue. The deregulation of IL-10–producing cells in dengue may thus contribute to the excessive inflammatory response observed during acute dengue infection [37].

Taken together, it seems that IL-10–producing B cells, by down-regulating CD8^+^ mediated immunity, aid to establish and/or maintain chronic viral infections such as HIV and hepatitis B in humans and herpesviruses in mice models [Table 1]. However, during acute infection, it seems that IL-10–producing B cells dampen the inflammatory response and are beneficial to the host. As described in the context of autoimmune diseases, it will be interesting to investigate more in detail which B cell subsets contribute to the production of IL-10 during acute and chronic viral infections.

### The role of other cytokines produced by B cells during viral infection

Lymphotoxins (LTα and LTβ) are members of the TNF superfamily. LT-α/-β signaling through the LTβ receptor is required for the establishment and maintenance of lymphoid structures [38]. Blockade of signaling through the LTβR results in decreased lymphocyte migration into lymph nodes (LNs). In B cells, LT is required for normal development of follicular DCs and is expressed as a secreted homotrimer, LTα3, or as a membrane-bound heterotrimer, LTα1β2. Only recently, it has been elucidated that the lymphotoxin signaling pathway also plays a critical role in the defense against viral infections by inducing the production of type I interferons (IFNs). The binding of LT-α/-β to LTβR initiates the differentiation of stromal cells and macrophages in lymphoid organs and rapid production of IFN-I in response to virus infections independently of the conventional TLR signaling systems [39]. The role of lymphotoxin signaling pathway in the induction of type I IFN responses has mostly been studied in herpesvirus infections. For instance, mice deficient for LTαβR signaling infected with MCMV fail to mount the initial part of a biphasic IFNα/β response. Here, LTβ-producing B cells were found to be important for promoting the initial IFNα/β response by LTβR-expressing stromal cells in the spleen [40]. Similarly, in another study by Moseman and colleagues, B cells were the primary source of LTα1β2, which helped in maintaining protective subcapsular sinus macrophages in draining LNs. Here, it was observed that mice with B cell–deficient LN or those with LTα1β2-deficient B cells displayed an aberrant phenotype of subcapsular sinus macrophages [41]. These defective macrophages were unable to produce type I IFN, which controls the infection of vesicular stomatitis virus in intranodal nerves [41]. LT-αβ production by B cells also leads to reorganization of the secondary lymphoid organs and helps in the mounting of an efficient immune response. Infection with lymphocytic choriomeningitis virus (LCMV) induced the growth and reorganization of the peripheral LNs, which was dependent on the LTαβ-LTβR pathway [42]. Adoptive transfer experiments revealed that virus-induced LN remodeling required LTα_1_β_2_-expressing B cells, indicating an important role for this B cell subset in the immune response to chronic viral infections [42].

B cell subsets are also known to produce pro-inflammatory effector cytokines, IL-6 and TNF-α, following engagement of BCR and CD40. In the context of dengue infections, a study by Lin and colleagues investigated cytokine production by B cells and showed that in vitro infection of B cells by dengue virus (DENV) induced the production of IL-6 and TNF-α [43]. Another study showed that B cells isolated from pediatric dengue patients spontaneously secreted TNF-α, IL-6, and IL-10, and supernatants from cultures of purified B cells induced activation of allogeneic T cells [44]. During HIV infection, the distribution of B cell subsets is perturbed, which has an impact on the cytokine secretion profile. Naive and memory B cells from healthy donors and HIV aviremic individuals secrete higher quantities of LT-α, IL-6, and TNF-α compared to viremic individuals [45]. In the context of severe acute respiratory syndrome coronavirus 2 (SARS-CoV-2) infections, IL-6 is thought to be involved in the cytokine storm following infection and treatment of patients with IL-6 inhibitors is currently under review [46]. However, if IL-6 is derived from B cells or other cells such as monocytes or lung epithelial cells remains to be understood [47–50]. In patients with agammaglobulinemia, who are unable to mount adaptive immune responses due to defects in B lymphocytes, a milder course of coronavirus disease 2019 (COVID-19) infection was observed compared to patients without agammaglobulinemia [51]. This observation has lead to the speculation that IL-6 produced by B cells may contribute to the cytokine storm causing increased inflammation. The authors suggested that B cell depletion therapies might be considered during COVID treatment [51].

Overall, the quantity and quality of effector cytokines produced by different B cell subsets is dependent on different environmental stimuli received by the B cells. B cell–derived lymphotoxin can aid in resolution of viral infections by optimizing type I IFN responses and pro-inflammatory cytokines such as IL-6 and TNF-α produced by B cells may contribute to enhanced inflammation and T cell activation during acute viral infections but could help to control chronic HIV infection [Fig 2]. However, the further characterization of cytokine secretion profiles of different B cell subsets and their functional relevance in the context of various acute and chronic infections remains to be elucidated.

### Granzyme B–producing B cells

B cells have also been shown to participate in antiviral immune responses through production and secretion of serine protease GrB, which has cytotoxic activity [11]. Although GrB is considered to be naturally produced by cytotoxic effector cells such as NK cells and cytotoxic T lymphocytes, recent studies have demonstrated that the combination of IL-21 and BCR engagement enables B cells to produce and secrete GrB, although this is not accompanied by perforin production [11]. GrB secreted by B cells can enter into the cytosol of virus-infected cell independent of perforin, through mannose-6-phosphate receptor or by fluid-phase endocytosis and thereby contribute to the intracellular control of the pathogen by killing of infected cells [11] [Fig 2]. Secretion of GrB by B cells has also been observed in the context of virus infections. For instance, B cells from healthy donors vaccinated against tick-borne encephalitis virus, hepatitis B, and rabies secreted GrB upon exposure to same viral antigens in vitro [52]. Kaltenmeier and colleagues observed increased frequencies of GrB-expressing B cells in the peripheral blood of HIV patients compared to healthy individuals. In this case, the differentiation of B cells into GrB-expressing B cells in vitro is driven by HIV Nef protein-activated CD4^+^ T cells from HIV patients in the presence of IL-21 (but not CD40L) [53]. These GrB-expressing B cells were able to suppress the proliferation of CD4^+^ T cells from HIV patients both in vivo and in vitro suggesting that this B cell subset may contribute to the aberrant T cell response during HIV infection. The presence of a GrB-expressing B cell subset was also confirmed in a macaque model with simian immunodeficiency virus (SIV) infection [54]. Higher frequencies of GrB-expressing B cells were observed in SIV-infected macaques compared to the uninfected group. In addition, their numbers inversely correlated with SIV viral load, suggesting their contribution in containing virus infection. Similar to their counterparts in HIV patients, GrB-expressing B cells were able to regulate CD4^+^ T cell responses in macaques [54]. Taken together, these studies identify a novel subpopulation of B cells, which produces GrB, an effector molecule usually produced by cytotoxic NK cells and CD8^+^ T cells, in response to infection. It is speculated that GrB producing B cells may contribute to antiviral responses in the acute phase of infection by mediating apoptosis of infected cells or by cleavage of viral proteins. On the other hand, in case of viruses targeting immune cells like in case of HIV and SIV infecting, their activity may affect the development of adaptive responses. The functional relevance of B cell–derived GrB remains to be investigated. These studies are complicated by the absence of consensus markers for GrB-producing B cells, the observation that these cells develop in vivo under conditions of high IL-21 but low CD40L and the fact that GrB-producing B cells are less numerous compared to other cells, which conventionally produce GrB such as NK cells and CD8^+^ T cells. Clearly, their precise role in viral infections is yet to be fully elucidated.

### Antigen presentation by B cells during viral infections

B cells by virtue of expressing cell surface major histocompatibility complex class II (MHC II) molecules can function as professional APCs for CD4^+^ T cells [Fig 2]. Antigen presentation by B cells also occurs in the context of germinal centers [55]. This process allows B cells in the germinal centers to effectively present antigens, which are recognized and captured by the BCR and is important for the selection and expansion of clones producing antibodies with higher affinity [56,57]. Compared to classical APCs (DCs and macrophages), which are highly endocytic in nature, B cells differ in their antigen-processing capabilities in that they can only take up and process the specific antigen recognized by its BCR. As a result, B cells can efficiently take up and present specific antigen to prime cognate CD4^+^ T cells and initiate the immune responses or induce their differentiation into follicular helper (T_FH_) cells [58]. Thus, antigen presentation by B cells to T cells is also important for the development of high-affinity antibodies against a particular antigen. The modulation of antigen presentation in DCs and macrophages by several viruses is well studied. Consequently, we know that viruses have evolved multiple strategies to down-regulate the expression of MHC molecules on DCs and macrophages, resulting in less efficient priming of the T cell–mediated immune response [59,60]. However, relatively little is known of their effect on antigen-presenting function of B cells. In murine models of viral infections, viral nonstructural proteins are shown to modulate the expression of MHC molecules and thus interfere in antigen presentation by B cells. For example, infection of susceptible mice with murine norovirus strains, namely MNV-1 and MNV-3, was found to regulate B cell–mediated antigen presentation by blocking the up-regulation of surface MHC molecules [61]. This resulted in impaired activation of cytotoxic CD8^+^ T cells, which are important in controlling acute norovirus infection. Minor structural protein VP2 of murine noroviruses also regulated B cell antigen presentation in a virus-specific manner [61]. Infection of B cell lines with murine noroviruses in vitro up-regulated the expression of MHC I and other costimulatory molecules. Moreover, ex vivo analysis of B cells from mice infected with Friend retrovirus (FV) showed that infected B cells up-regulated the expression of costimulatory molecules CD80, CD86, and CD40, as well as MHC II molecules [62]. This research showed that in vitro infected B cells had significantly enhanced APC function, as measured by their capacity to prime CD8^+^ T cell activation and proliferation, compared to uninfected B cells from the same mice [62]. In a study involving human patients infected with thrombocytopenia syndrome virus (SFSTV), a bunyavirus, expression of HLA-DR and costimulatory molecule CD80 on B cells was significantly down-regulated in deceased patients compared to surviving patients [63]. Proportion of CD80^+^HLA-DR^+^ cells also differed between the deceased and the surviving groups, suggesting that SFSTV infection interferes with antigen presentation by B cells, which aggravates disease [63].

Some studies demonstrate that antigen-specific B cells loaded with viral peptides can act as professional APCs and thus may be used as a part of cell-based immunotherapy to stimulate antiviral T cell immunity [64–66]. For example, transgenic B cells expressing immunodominant peptides from influenza nucleoprotein were injected and used to prime mice before live influenza virus challenge. These transgenic B cells induced effective protective responses from memory CD8^+^ T cells against the virus challenge even in the absence of DCs [64]. Similarly, another study showed that CD40-activated B cells electroporated with mRNA for cytomegalovirus or influenza nonstructural proteins and/or pulsed with viral peptides were able to reactivate CD4^+^ and CD8^+^ memory T cells from human peripheral blood mononuclear cells (PBMCs) with CD8^+^ T cells showing increased IFN-γ production. Interestingly, no difference was observed between the antigen presentation capacity of B cells and monocyte-derived DCs from the same donors [65]. Hong and colleagues used virus-like particles as an antigen and showed that antigen-specific B cells were the dominant APCs that initiated the activation of naive CD4^+^ T cell and induced the development of Tfh cells even in the absence of DCs. Antigen-specific B cells were also involved in initiating CD4^+^ T cell responses during immunization with inactivated influenza virus [66].

B cells offer several potential advantages over DCs in cell-based immunotherapy or vaccination strategies. B cells are numerous in peripheral blood (approximately 10% of lymphocytes) compared to DCs. Hence, it is easier to obtain sufficient numbers (approximately 1 × 10^5^ to 1 × 10^7^ cells/kg body weight) of activated antigen-presenting B cells [67], whereas the generation of DCs typically requires a leukapheresis [63]. It has been shown that this is even feasible in cancer patients [67]. Activated B cells can serve as APCs for both CD4^+^ and CD8^+^ T cells. The prime advantage of B cells compared to DCs is that they can selectively present the cognate antigen collected through surface Ig molecules, even at a minimal concentration of Ag [67].

Overall, using the function of B cells as APCs may serve as a useful alternative for DCs in cell-based immunotherapy or vaccine strategy against viral infections.

### B cell responses after viral infection/stimulation via TLRs

It is well established that stimulation of mature B cells in vitro with TLR ligands leads to proliferation and differentiation of B cell into plasma cells. TLR expression and their activation profiles are different between human and mice, with the most prominent differences in TLR2 and TLR4, which has been extensively reviewed by Bekeredjiang-Ding and Jego [68,69]. While some studies have investigated TLR2- and TLR4-mediated responses to viral infections using murine B cell models, similar studies have not been performed in human B cells as they express little or no TLR2/4 [70]. Both mouse and human B cells express endosomal TLRs, such as TLR3, which binds dsRNA, TLR7/8, which senses single-stranded RNA, and TLR9, which binds CpG-containing DNA [71]. TLR7/9 engagement leads to NF-kB–dependent inflammatory cytokine production and type I IFN production [71]. The roles of TLR7 and TLR9 in B cells and their crosstalk with BCR ligation have been extensively studied in models for systemic lupus erythematosus (SLE) [72–76]. Intrinsic TLR7 and TLR9 signalling in B cells has been shown to play an important role in the development and pathogenesis of SLE. While TLR7 activation is critical for the extrafollicular and germinal centre responses associated with the activation of autoreactive B cells, TLR9 engagement could have beneficial functions in patients with SLE by restraining TLR7 function. Recently, B cells have recently been shown to produce type I IFN in vivo after optimized stimulation conditions using the TLR9 ligand CpG-A [77]. However, the role of TLR7/9 stimulation in B cells during viral infections remains to be elucidated.

Although B cells can be activated with endosomal TLR ligands through nonspecific uptake in vitro, TLR signaling in synergy with BCR activation is far more potent in stimulating B cell activation. TLR signaling in B cells plays an important role in the antibody response and is mainly dependent on the expression of MyD88, a key adaptor protein in the TLR pathway [78]. Mice deficient in MyD88 have been shown to have reduced antibody responses to immunizations and viral infections [79–81]. A study by Hong and colleagues concluded that MyD88 signaling mediated by viral-like particles (VLPs) in B cells is required for the induction and the development of T_FH_ cells to specific antigens. Here, antigen-specific CD4^+^ T cells transferred to B cell–specific MyD88^−/−^ mice exhibited a defect in the up-regulation of T_FH_-associated signature molecules [66,82]. Altogether, these observations suggest that TLR-mediated B cell responses contribute to the differentiation of CD4^+^ T cells into T_FH_ cells that in return are crucial for the development of virus-specific humoral immunity. Indeed, several studies in mouse models of FV and LCMV infections have highlighted that TLR7 signaling in B cells was important in mediating antiviral responses and that loss of TLR7 correlated with impaired germinal center response [83–85]. Stimulation of both human or mouse B cells with TLR ligands alone leads to increased cellular responses, antigen presentation, proliferation, class switch recombination, and antibody secretion and up-regulation of activation markers CD69, CD80, and CD86 [16,17,86,87]. However, human B cells isolated from dengue patients showed a different response upon stimulation with TLR ligands. When stimulated with TLR9 agonist CpG, we have shown that activation markers CD69 and CD86 were not up-regulated and that no secretion of IL-10 and TNF-α was observed in B cells from dengue patients compared to healthy donors. However, when stimulated with CpG in conjunction with BCR ligation, these cells showed an activated phenotype, showing that B cells from dengue patients are refractory to sole TLR9 stimulation [37]. It remains to be investigated if this is due to lower expression of TLR9 in B cells from dengue patients or if DENV interferes with the TLR signaling pathway in B cells. The effect of TLR9 stimulation with CpG on B cell responsiveness has also been studied in HIV patients. In parallel with the findings in dengue infection, B cells from healthy individuals and chronic HIV patients did not produce significant quantities of cytokines upon stimulation with CpG alone [45]. However, when combined with BCR and CD40 ligation, higher amounts of cytokines were detected. In both the case of HIV and DENV, the memory B cell compartment seems terminally differentiated and therefore unresponsive to TLR stimulation alone.

To summarize, TLR signaling in B cells plays an important role in the development of a potent humoral immune response to viral infections by promoting the differentiation of CD4^+^ T cells into T_FH_ cells. Furthermore, upon TLR stimulation, B cells are activated and show enhanced antigen presentation and production of effector cytokines [Fig 2]. However, it seems that viruses have evolved strategies to subvert TLR signaling in B cells or TLR-mediated B cell responses, and B cells may require ligation of BCR as an additional stimulus to overcome these deficiencies.

### The role of inhibitory receptors on B cells during viral infections

B cells become activated due to the binding of antigen to the BCR, which induces BCR clustering and triggers downstream positive signaling. The activation signals are counterbalanced by negative signals, which are generated by membrane receptors (PD-L1, CD22, CD72, FcγRIIb, paired Ig-like receptor B [PIR-B], and LILRB1). A recent study by Epeldegui and colleagues showed that frequencies of Bregs-expressing programed death-ligand 1 (PD-L1), which binds to PD-1, were elevated in patients with chronic HIV infection prior to the appearance of non-Hodgkin lymphoma associated with HIV-AIDS [88]. Furthermore, it has been shown that during HIV infection, Bregs activated via TLRs cells up-regulate PD-L1 and can inhibit CD4^+^ T cells through both IL-10 and PD-L1 [28,89]. Therefore, expression of PD-L1 on B cells may provide additional pathway for Bregs cells to inhibit beneficial anti-HIV T cell responses. A similar observation was made in PBMCs infected in vitro with varicella zoster virus where infected PBMCs, including B cells, showed significant elevations in PD-L1 and PD-L2 expression compared to uninfected cells. The authors hypothesize that this elevated PD-L1 may bind to PD-1 on CD8^+^ T cells leading to immune suppression and establishment of VZV infection in patients [90]. In the context of dengue infection, we observed increased expression of inhibitory receptors CD32B and LILRB1 on CD19^+^CD27^−^ naive B cells during acute dengue infection. As a consequence, these cells showed decreased activation and cytokine production after TLR/anti-IgM stimulation in vitro, which might play a role in dengue pathogenesis [37].

Thus, overall, the expression of various inhibitory receptors on B cells may represent an important mechanism in regulating the immune responses to viral infections and needs to be investigated further.

### Conclusions and future perspectives

B cells have diverse antibody-independent functions, such as production of regulatory and pro-inflammatory cytokines, antigen presentation, and are responsive to stimulation with TLR ligands. These functions intersect with components of both innate and adaptive immunity and help in shaping the immune response to autoimmune diseases, infections, and tumors. In this review, we summarized the antibody-independent B cell responses in the context of acute and chronic viral infections (Fig 2). We focused on the IL-10–mediated regulation of the immune response by various B cell subsets (including Bregs), production of other cytokines by B cells, antigen presentation, and TLR-mediated activation of B cells during viral infections.

Bregs constitute a small population of B cells that participates in immunomodulation and suppression of immune responses. They regulate the immune system through different mechanisms, primarily through the production of anti-inflammatory cytokine IL-10. The regulatory effects of Bregs have been described in the context of inflammation, autoimmune diseases, transplantation, and antitumor immunity. During acute and chronic viral infections, it was observed that production of IL-10 by Bregs had both beneficial and detrimental effects in the pathogenesis of disease. The depletion of B cells with rituximab has shown potential in the treatment of autoimmune diseases [91,92]. However, removal of the entire B cell population may lead to reactivation of latent virus infections [93] or render the patients susceptible to opportunistic infections [94–96]. Therefore, depletion therapies targeting specific B cell subsets, rather than all B cells, should be considered. For instance, in situations where Bregs contribute to exacerbation of viral infections by suppressing beneficial CD8^+^ T cell–mediated immunity, such as in patients with HBV/HIV infections, depletion of B cells using rituximab might have a beneficial effect. Therefore, further studies will be needed to define the phenotype of B cell subsets involved in IL-10 production during viral infections as currently no specific marker is available for the many B cell subsets that can produce IL-10.

Since B cells can act as APCs, they may also serve as a useful alternative for DCs in cell-based immunotherapy or vaccine strategies against viral infections. There is evidence that B cells, when stimulated by cross-linking of the BCR by cognate antigen and ligation of CD40 with CD40L on activated T cells, up-regulate MHC II and CD80/CD86. Currently, there are few preclinical and clinical studies in mice, dogs, and humans utilizing the potential of these CD40-stimulated B cells as APCs as a part of cancer immunotherapy [67]. Another promising approach for developing viral vaccines could be using TLR7, 8, and 9 ligands as adjuvants, which stimulate naive B cells, induce the production of antibodies and promote cellular differentiation into long-lived plasma cells [17,97,98]. Currently, there are several successful TLR agonist-adjuvanted vaccines for cancer and viral infections that have been originally designed to target myeloid APCs. It is important to note, however, that these vaccines may have unwittingly influenced antigen presentation by B cells. B cells activated via TLR up-regulate activation markers and produce pro-inflammatory cytokines, which can enhance vaccination response. However, the contribution of B cell in the efficacy of the TLR-adjuvanted vaccines remains to be investigated.

In conclusion, B cell exert antibody-independent functions that seem to play an important role in resolution or exacerbation of viral infections. Importantly, a deeper understanding of these B cell functions in different acute and chronic viral infections will enable their utilization as basis of novel therapeutic approaches and vaccination strategies.
